# Is More Always Merrier? Intersectionality as an Antecedent of Job Insecurity

**DOI:** 10.3390/ijerph15112559

**Published:** 2018-11-15

**Authors:** Lindsey M. Lavaysse, Tahira M. Probst, David F. Arena Jr.

**Affiliations:** 1 Department of Psychology, Washington State University Vancouver, 14204 NE Salmon Creek Avenue, Vancouver, WA 98686-9600, USA; probst@wsu.edu; 2 Department of Management, University of Memphis, 3675 Central Avenue, Memphis, TN 38152, USA; dfarena@memphis.edu

**Keywords:** intersectionality, job insecurity, diversity, economic stress, occupational health

## Abstract

As modern workplace environments are becoming increasingly diverse, the experiences of disenfranchised employees have become a topic of great interest to scholars and business professionals alike. While the experiences of individuals with singular stigmatized identities have been well-established, a dearth of research has assessed how intersectionality, i.e., holding multiple stigmatized identities, combine and intertwine to shape workplace experiences. We contribute to a growing literature on intersectionality by assessing the extent to which employees identifying with multiple stigmatized identities may constitute a risk factor for the experience of job insecurity, a prevalent and potent economic stressor. Additionally, we propose that job insecurity will partially mediate the relationship between intersectionality and a variety of adverse workplace outcomes associated with increased job insecurity perceptions. In order to test these hypotheses, we collected survey data from 449 employed individuals within the United States over two timepoints. Results of the tests of our direct and indirect hypotheses revealed that individuals with more stigmatized identities reported greater perceptions of job insecurity, and intersectionality indirectly affected workplace outcomes via this heightened job insecurity. Our results highlight a new antecedent of job insecurity for consideration and is meant to motivate others to approach diversity-related research questions with multiple identities in mind, in an effort to encapsulate the full spectrum of one’s experience based on their identity makeup.

## 1. Introduction

Respect, fairness, and job security are verifiable factors that human resource professionals can emphasize and promote to maintain employee motivation [1]. In response to an ever-changing and rapidly diversifying workforce, scholarship surrounding some of the above areas has skyrocketed to discern how to effectively manage diversity and promote a safe and inclusive workplace for individuals with marginalized identities. While research has effectively documented the relationship between perceptions of employee justice or fairness and positive workplace related outcomes (i.e., commitment and intentions to quit [2]), there has been a significant dearth of work focused specifically on job insecurity. Job insecurity represents a salient economic stressor that relates to a variety of negative work-related outcomes verified by both longitudinal [3] and meta-analytic [4] work. In a tumultuous economic environment, employees may be more attuned to the idea that steady work may not be constant, which can relate to detriments to employee health and well-being [5]. Indeed, some scholars highlight the overall negative impact of increased job insecurity on employee job attitudes, health and well-being, and their overall relationship with their organization [4,6,7].

Although there has been an uptick of research in this area following the ‘Great Recession’ of 2007–2008, the bulk of this has typically focused on the consequences of job insecurity such as apparent threats to employee physical and mental health, safety, performance, job satisfaction, and turnover intentions [4,6,7]. While the outcomes of increased job insecurity are, without doubt, important, we argue that an imperative extension of previous work involves an assessment of the factors that may precede job insecurity—especially those associated with the identity characteristics of employees. Regardless of the current socio-political discourse surrounding the importance of workplace diversity and the value of egalitarianism, there is next to no direct and empirical research focused on how the experience of job insecurity may differ as a function of demographic or identity characteristics. Indeed, while scholars have found that diversity-focused research is on the rise in top tier research outlets [8], there has been scarce work assessing the nuanced experience of job insecurity in employees with stigmatized identities. We are aware of one meta-analysis [9] that evaluated how age and gender influenced job insecurity, but this study assessed these variables independently of other identities and did not consider other relevant identities of marginalized employees. We argue that the average employee is likely to possess more than one stigmatized identity, and as such, assessing identities individually may not be enough to assess the factors that may increase one’s vulnerability to job insecurity.

As the workforce is becoming increasingly diverse and intersectional [10], we argue that an important extension of past work on employee experiences of job insecurity comes from the lens of intersectionality [11], i.e., the experiences of employees who identify with multiple minority status identities (for example, an individual who identifies as both female and Latina or an individual who identifies as African American, Muslim, and homosexual). Integrating diversity and occupational health literatures, a primary goal of this paper is to assess how combinations of multiple stigmatized identities—intersectionality—may make one vulnerable to job insecurity perceptions, and by extension, the known health and work-related outcomes of this economic stressor.

Taken together, our study contributes to existing organizational scholarship in two primary ways. First, we broaden past work on job insecurity by assessing the demographic makeup of employees—intersectionality—as a meaningful antecedent. In doing so, we integrated foundational arguments from intersectionality-focused research [11] with modern job insecurity frameworks to articulate how individuals with multiple minority status identities may experience feelings of job insecurity as they navigate their professional lives. The results of our study contribute to a growing body of work highlighting the unique workplace experiences of multiple minority status individuals [12], and identify a meaningful antecedent to a well-established economic stressor. We further explore this phenomenon by exploring how intersectionality could indirectly influence job-, health-, and work-related outcome variables through heightened job insecurity. As such, we advance several mediation models wherein intersectionality precedes previously established relationships between job insecurity and workplace outcomes.

Second, we advance a new way of conceptualizing intersectionality by measuring this variable additively—adding methodological nuance to our paper. We assessed identities that were both protected (i.e., race or gender) as well as unprotected (i.e., sexual orientation) by federal legislation in the United States, to encapsulate a greater spectrum of the identity makeup that any employee may possess. Past scholars have argued that “as the number of multiple minority statuses increases, the likelihood of decreased social and economic resources also increases” [13] (p. 40), lending credence to our decision to study this variable additively. While the results of our manuscript cannot speak to the overall impact of identifying a specific combination of stigmatized identites, we can extrapolate as to how identifying with a greater number of stigmatized identities can influence job insecurity.

We begin by detailing our contextual definition of ‘intersectionality’. Next, we discuss past work on intersectionality to provide support for the nuanced experience of individuals who possess multiple minority statuses at work before addressing past work on economic stressors and job insecurity to ground these ideas in a workplace context. We then propose several mediation models wherein job insecurity mediates the impact of employee intersectionality on previously established outcomes of job insecurity. Our study contributes to the overarching literature on workplace diversity and economic stressors by providing a better understanding of how the multiple intersecting identities that any employee holds and carries throughout their work life may impact their health, safety, and well-being through their perceptions of job insecurity.

### 1.1. Intersectionality Defined

According to the U.S. Equal Employment Opportunity Commission, it is illegal to discriminate against an employee (or applicant) on the basis of an individual’s race, color, religion, sex (including pregnancy, gender identity, and sexual orientation), national origin, age (specifically, 40 or older), disability, as well as being a covered military veteran. Nearly all of the states in the U.S. include marital status as a protected class as well. The laws put into place to protect stigmatized groups within these classes were due to their historical experiences of discrimination in the workplace.

Since the inception of the term “intersectionality” by Crenshaw [11], research in a variety of disciplines has exploded to explore this new dimension of diversity-related work. Originally conceptualized as a method for addressing the inadequacy of federal legislature in attenuating the discrimination faced by Black women [11], intersectionality has evolved to capture any given intersection between multiple stigmatized identities. More recent evidence suggests that protection for intersectional employees is still not adequate, as individuals who filed intersectional claims with the Equal Employment Opportunity Commission (EEOC) were only half as likely to win as compared to those who filed a claim on a single basis of discrimination [14]. As dialogue considering the experiences of marginalized employees continues to be a topic of importance to both scholars and business professionals, a conversation has begun on how best to assess research topics through an intersectional lens.

Crenshaw [11] advanced the notion of double discrimination, to encapsulate how Black women sometimes experience forms of discrimination similar to that of white women and that of Black men in different settings. This specific scenario highlights that there are two types of discrimination at play, both race-related and gender-related, which are distinct but intertwine with each other to the shape experiences of Black women. This example highlights that different and unique identity characteristics can individually contribute to the experiences of intersectional employees. A distinguishing factor in modeling intersectionality continues to be whether identities interact additively (unique identities sum together to produce a nuanced experience) or multiplicatively (unique identities interact to produce an overall nuanced experience [15]). Simply, the additive approach considers identities as “static at the individual or institutional level” whereas the multiplicative approach considers identity status as a “dynamic interaction between individual and institutional factors” [16] (p. 64). Hancock [16] continues to discuss how the relationship between stigmatized identities—she focuses on race, gender, class, and “other categories”—differs based on the approach as well. In an additive approach, the identities matter equally in reference to each other, while in multiplicative approaches the relationship between categories remains an answerable question throughout the research process.

While there is debate as to which of these methods for understanding and conceptualizing intersectionality is more methodologically rigorous, we argue that both approaches merit future research. For the purposes of this paper, we decided to assess intersectionality as an additive metric such that we coded identities based on whether they were characterized as ‘stigmatized’ or ‘non-stigmatized’. As such, we define intersectionality in our paper as the overall sum of stigmatized identities (defined by employment protection legislation and prior empirical research). As such, our additive measure of intersectionality was developed as a function of identifying with one or more of the stigmatized groups of employees within the following classes: gender, race, age, sexual orientation, military status, marital status, religious affiliation, and nationality. Our method section provides more detail on our measure of intersectionality.

### 1.2. Past Work on Intersectionality

‘Intersectionality’ has become a buzzword in recent years [17], even appearing in popular press articles [18]. Considering this, scholarship has moved to discern the boundary conditions of intersectionality theory. This work has linked intersectionality with other prominent theoretical areas [19] including feminism [20], social justice [21], and humanities such as anthropology and sociology [22]. As is common practice for the study of a phenomenon with a dearth of foundational work, scholars conducted a good amount of qualitative research in the past several years assessing the experiences of intersectional employees. Indeed, Warner [23] discusses that community-based qualitative research is an effective way to assess the nuanced experiences of intersectional employees—and research has followed this recommendation. For example, Logie and colleagues [24] conducted a qualitative focus group study to understand the experiences of HIV-positive women in Canada. This study found support for multiple levels of stigma associated with race, gender, sexual orientation, and HIV-status that combined to influence health and well-being. Another study conducted by Mattis and colleagues [25] discerned the differing vulnerability of participants in a qualitative study based on gender, race, ethnicity, class, age, and urbanicity. Furthermore, Bowleg [26] applied a qualitative approach to understand the experiences of gay and bisexual men of color. While qualitative research is relatively abundant, there has been significantly less quantitative work on intersectional employees (beyond the intersection of two or three identities). For example, one study assesses the intersection of race and gender in survey research using archival survey data and calls for future scholars to develop better survey measures to assess intersecting identities and prejudices [27]. As research in this area develops, we can say with confidence that the amount of stigmatized identities shapes the experiences that people have—although there is debate as to whether intersectionality has a positive or negative influence on outcomes [28].

### 1.3. Intersectionality in Workplace Settings

As current socio-political discourse dictates, discrimination pervades everyday organizational environments. Adding to the complexity of grappling with discriminatory treatment at work is the changed nature of workplace discrimination away from more overt manifestations and toward subtle and ambiguous expressions of interpersonal mistreatment [29]. There has been a great amount of research in the past decade focused on how marginalized populations navigate workplace environments, and how the experiences of marginalized populations differ from those in the majority. Evidence suggests that disenfranchised populations such as women [30], racial minorities [31], religious minorities [32], sexual orientation minorities, [33] and older employees [34] endure different forms of mistreatment at work. For example, past work details that marginalized employees are perceived as incompetent [35] and perceive greater instances of both formal and interpersonal discrimination from others as compared to majority status employees [36]. This perceived or recognized interpersonal mistreatment at work relates to a variety of negative job-related outcomes such as lower commitment, lower job satisfaction, greater work tension, less engagement, higher withdrawal, greater depressive symptoms, and greater job insecurity [35,37,38,39,40].

While this work moves us forward in understanding the experiences of individuals with stigmatized identities, a good bit of diversity-related organizational research focuses only on one stigmatized identity per study—a limitation that highlights the need for workplace research conducted through the lens of intersectionality. Indeed, Rodriguez and colleagues [41] state that “…despite its robust potential, intersectionality remains at the margins of dominant work and organization narratives of equality and inclusion” (p. 202). In response to this, there has been some semblance of research focused on the intersection of multiple identities arguably applicable to workplace settings. For example, Zurbrügg and Miner [42] found that sexual minority women reported greater perceptions of workplace incivility as compared to all possible other combinations of the intersection between gender and sexual orientation. Additionally, Bowleg and colleagues [43] found evidence of a triple-jeopardy experience of Black lesbians wherein they reported stress associated with multiple minority statuses (however, these women also reported greater resilience to racism). This minority stress can evoke aggressive behavior at work [44] and counterproductive work behaviors [45]—both of which will alter any given employee’s status at work.

While there has been no direct work assessing the effect of intersectionality on job insecurity, we find that minority stress theory [46] provides an avenue for explaining this relationship. Scholars define minority stress as the psychosocial stress that marginalized individuals inherently possess derived directly from their minority status [46,47]. As enduring discrimination at work and an awareness of societal stereotypes pertaining to one’s identity makeup influence minority stress, this theory has become a prominent theoretical foundation for diversity-related research contextualized at work. Indeed, scholars use minority stress theory to explain the workplace experiences of individuals with singular stigmatized identities [48,49,50] and double jeopardy identities [51,52]. The key tenet of these studies applicable to intersectionality is that as stigmatized identities combine and intertwine, stigmatized employees feel increasingly stressed about their work situations and may be more apprehensive about their longevity at any given organization.

Arguments surrounding minority stress theory are germane to explaining how identifying with multiple stigmatized identities may relate to greater job insecurity. Past scholars provide compelling arguments as to why adding another layer to one’s overall stigmatized identity may lead to increased workplace mistreatment [53], and through this, greater work-related stress. Accordingly, we argue that individuals who identify with a greater number of stigmatized characteristics may incur more workplace stressors due to being targeted more frequently by workplace harassment aimed at different identity statuses. As such, intersectional employees are likely to feel uneasy about their status within their organization and may indicate greater perceptions of job insecurity as compared to those who identify with fewer stigmatized identities. Supporting this notion broadly, Camgoz and colleagues [54] found differential gender effects for concerns about losing one’s job privileges (but not the job itself). Kuroki [55] found that African American workers had higher mean job insecurity than Caucasian workers in an analysis of 35 years of the American General Social Survey (GSS). Finally, Wilson and Mossakowski [56] found that African American and Latino men and women indicated a greater level of job insecurity as compared to White men and women. While this work provides preliminary evidence of the different experiences of individuals with different identity makeups on job insecurity, they fail to encapsulate how the intersection of identities may influence this experience. Considering this, we integrated minority stress theory [46] to argue that individuals with a greater number of stigmatized identities will report greater feelings of job insecurity as their identities intertwine to influence their workplace experiences. Based on this theoretical basis, we predict that:

**Hypothesis 1** **(H1).** Individuals who identify with more stigmatized identities will report higher perceived job insecurity at both Time 1 and Time 2.

### 1.4. Intersectionality Impacting Occupational Health and Safety via Job Insecurity

Past work assessing heightened perceptions of job insecurity has revealed the negative health-, interpersonal-, and work-related impact of the feelings of uncertainty associated with one’s employment. In fact, the results of several meta-analyses highlight the severity of job insecurity. For example, Sverke and colleagues [7] meta-analyzed 72 studies wherein they found that job insecurity related to lower job satisfaction, lower job involvement, lower organizational commitment, and lower health outcomes. Cheng and Chan [6] extended this work by assessing 133 studies wherein they found a significant relationship between job insecurity and increased turnover intentions, lower job performance, and replicated the results of Sverke and colleagues [7] on several other meaningful outcome variables. Furthermore, a recent meta-analysis by Jiang and Lavaysse [4] extended the previous research even further by incorporating 41 outcome variables and 14 correlates of job insecurity, while simultaneously exploring the validity of distinguishing between cognitive and affective components of job insecurity. There is also evidence to suggest that job insecurity may relate to decreased safety behaviors. For example, Probst and Brubaker [57] found that heightened job insecurity led employees to display lower safety motivation and safety compliance. The above research is evidence that increased job insecurity has a variety of negative work-, health-, and interpersonally-related outcomes on employees. We intend to replicate some of these results in our study by showing that heightened job insecurity relates to a variety of negative outcomes (i.e., lower safety motivation, safety compliance, affective commitment, normative commitment, continuance commitment, job satisfaction, physical health, mental health, and heightened turnover intentions).

Indeed, the relationship between job insecurity and a variety of negative work and health-related outcomes is salient. Considering the foundational work conducted on the outcomes of heightened job insecurity, we advanced this work to assess intersectionality as a preceding factor to previously determined relationships, extrapolating on how intersectionality related to known outcomes of increased economic stress through heightened job insecurity. Moreover, we argue that the economic stress associated with increased job insecurity could partially explain the relationship between identifying with a greater frequency of stigmatized identities and several work-related outcomes. Extant scholarship has identified that workplace-related stressors are a salient factor in eroding the job attitudes of minority employees. For example, Lehavot and Simoni [58] found that sexual minority women (a double jeopardy identity status) reported more mental health problems through increased minority stress (operationalized through heterosexism). Williams and colleagues [59] found racial differences in both physical and mental health through differences in economic and non-economic stress. Broadly, there is an abundant amount of evidence for the negative work-related outcomes for those susceptible to discrimination (according to extant theory, individuals who identify with greater stigmatized identities may endure greater frequencies of discrimination) at work such as increased turnover intentions, lower organizational commitment, lower job satisfaction, and lower mental and physical health [60,61]. Thus, there is evidence to suggest that minority status employees may have a different experience at work as compared to their non-stigmatized identity status counterparts.

The above arguments present a scenario wherein employees with more stigmatized identities endure stress at work that permeates into their job attitudes and behaviors. According to minority stress theory [46], minority status individuals are privy to psychosocial stress at work based on their identity status. Taking an intersectional spin [11] on this perspective yields that individuals with greater stigmatized identity statuses may endure greater amounts of stress, which may erode relevant job attitudes. Indeed, Kӧllen [62] expanded on this idea in presenting a theoretical model of understanding how minority status employees manage stress at work. He related how different identities are perceived in the larger organizational hierarchy to argue that employees may be grappling with minority stress from more than one identity at a time. While work in this area is in its infancy, we argue that economic stress may act in the same way as psychosocial stress, such that as identities intertwine and compile, stress will increase, and organizationally relevant variables such as commitment, turnover intentions, and employee well-being will be negatively eroded. Thus, increased economic stress or job insecurity could act as a partial mediator between intersectionality and work-related outcomes. Together, we first expect to replicate previous findings as to the damaging effects of heightened job insecurity. We then propose several partial mediation models to test how intersectionality could relate indirectly to multiple known outcomes of job insecurity through heightened perceptions of job insecurity. Formally, we predict that:

**Hypothesis 2** **(H2).** Heightened job insecurity will relate to lower safety motivation, safety compliance, affective commitment, normative commitment, continuance commitment, job satisfaction, physical health, mental health, and heightened turnover intentions.

**Hypothesis 3** **(H3).** Intersectionality will have an indirect relationship on previously established outcomes of job insecurity (i.e., safety motivation, safety compliance, affective commitment, normative commitment, continuance commitment, turnover intentions, job satisfaction, physical health, and mental health) through heightened job insecurity.

## 2. Materials and Methods

### 2.1. Participants and Procedure

Participants were recruited through Amazon’s Mechanical Turk, an online recruiting platform for human subject research. Due to the anonymous nature of the data collected, the study was considered exempt by the first and second authors’ Institutional Review Board (Review Category: Exempt—45 CFR 46.101 (b)(2); Certification Number: 16090-001).

In order to test our hypotheses, we recruited working adults over the age of 18 with the stipulation that participants were employed outside of Amazon’s Mechanical Turk and resided in the United States. To better ensure the collection of high quality data, researchers recommended only recruiting MTurk workers with a high reputation [63], which was attained by providing quality data on previous tasks in order to develop an established positive track record. Thus, we required a minimum of a 95% prior approval rating across a minimum of 500 previously completed tasks. Additional quality control checks were utilized throughout the survey. Specifically, two questions scattered throughout inquired as to whether the individual was truly reading each item (e.g., If you are reading this, please select “Agree”). No further limitations to the recruiting process were used as we aimed to have as diverse a demographic make-up of the group as possible.

Data were collected at two time points with a one-week temporal lag, a strategy recommended for reducing mono-method bias issues [64]. The final dataset included N = 502 at Time 1 and N = 449 at Time 2, yielding roughly a 10% attrition rate—which is to be expected when conducting survey research with more than one timepoint. Our sample included 283 men and 214 women (M_age_ = 34.79). Additionally, our sample was primarily heterosexual (84%), Caucasian (70%), and identified as single (50%).

Consenting participants were invited to complete a two-wave survey (Time 1 and one-week follow-up) assessing key study variables (e.g., perceived job insecurity, safety, commitment, turnover, job satisfaction, and mental and physical health). Specifically, intersectionality and perceived job insecurity were assessed at T_1_, and one week later, T_2_: job insecurity, safety, commitment, turnover intentions, job satisfaction, and mental and physical health.

### 2.2. Measures

#### 2.2.1. Intersectionality

Participants provided their race, marital status, gender, sexual orientation, military status, age, religion, and nationality, so that we could assess how many stigmatized identities each participant possessed. Based on employment protections and/or numerical representation at work, non-stigmatized identities included: male, white, married, heterosexual, non-veteran, under 40 years of age, Christian, and a U.S. citizen. Intersectionality was calculated as the sum of stigmatized identities for each participant. For example, a 57 year old female (both stigmatized) with non-stigmatized identities for the remainder of the demographic questions received an intersectionality value of 2. Thus, higher values indicated more stigmatized identities. Among our sample of respondents, observed values ranged from 0 to 6.

#### 2.2.2. Job Insecurity

The Job Security Index, a 9-item measure by Probst [65], assessed perceived job insecurity of employees. Respondents indicated on a 3-point scale (yes, don’t know, no) the extent to which each item described their perception of the future of their job. A sample item included, “can depend on being here”. Responses were scored such that higher numbers reflected more job insecurity. Based on findings suggesting that endorsement of the “don’t know” anchor was psychometrically closer to a negative response than a positive one [66], the scoring system recommended by Hanisch [66] was utilized. Specifically, the item responses were coded as follows: agreement with negatively worded items (i.e., “unknown”) was scored 3; agreement with positively worded items (i.e., “stable” and “can depend on being here”) was scored 0; and “don’t know” responses were scored 2.

#### 2.2.3. Safety Motivation

To assess safety motivation at work, a 4-item scale was developed by Neal, Griffin, and Hart [67], using a 7-point Likert scale from strongly disagree (1) to strongly agree (7) to gauge one’s level of agreement. A sample item included “I believe that it is important to reduce the risk of accidents and incidents in the workplace”.

#### 2.2.4. Safety Compliance

An 8-item scale developed by Neal, Griffin, and Hart [67] was used to assess safety compliance in the workplace. One’s level of agreement was reported using a 7-point Likert scale from strongly disagree (1) to strongly agree (7). A sample item included, “I carry out my work in a safe manner”.

#### 2.2.5. Affective Commitment

An 8-item scale used a 7-point scale from strongly disagree (1) to strongly agree (7) to assess the employee’s affective commitment [68]. A sample item included, “I enjoy discussing my organization with people outside it”.

#### 2.2.6. Continuance Commitment

An 8-item scale used a 7-point scale from strongly disagree (1) to strongly agree (7) to assess the employee’s continuance commitment [68]. A sample item included, “too much in my life would be disrupted if I decided I wanted to leave my organization now”.

#### 2.2.7. Normative Commitment

An 8-item scale used a 7-point scale from strongly disagree (1) to strongly agree (7) to assess the employee’s normative commitment [68]. A sample item included, “I do not believe that a person must always be loyal to his or her organization”.

#### 2.2.8. Turnover Intentions

A 3-item scale developed to measure turnover with a 5-point Likert scale was used [69,70]. A sample item included “how often do you think about quitting your job?”.

#### 2.2.9. Job Satisfaction

A 3-item scale used with a 7-point Likert scale from strongly disagree (1) to strongly agree (7) by Cammann, Fichman, Jenkins and Kelsh [71] assessed the participants general job satisfaction. A sample item included “all in all, I am satisfied with my job”.

#### 2.2.10. Physical Health

A 13-item scale developed by Brodman, Erdman, Lorge, Wolff and Broadbent [72] used a 3-point scale (yes, maybe, no) to assess the employee’s physical health. A sample item included, “respiratory or lung problems”.

#### 2.2.11. Mental Health

A 15-item scale used a 6-point scale from none of the time (1) to all of the time (6) to assess the employee’s mental health [73]. Participants were asked to report the frequency of occurrences within the last month. Some sample items included, “how often have you felt tense or ‘high strung’?”, “how often have you felt emotionally stable?”, “how often did you find yourself having difficulty trying to calm down?”.

## 3. Results

### 3.1. Descriptive Statistics and Scale Intercorrelations

Table 1 presents the descriptive statistics, scale reliabilities, and intercorrelations among the study variables at the two time-points. As predicted by Hypothesis 1, intersectionality was significantly and positively associated with job insecurity at both Time 1 (*r* = 0.13, *p* < 0.01) and Time 2 (*r* = 0.12, *p* < 0.05). Consistent with previous research, job insecurity (measured at T1) was significantly and negatively correlated with subsequent T2 measures of: safety motivation and compliance (*r* = −0.13 and *r* = −0.14, respectively, *p* < 0.01); affective, continuance, and normative commitment (*r* = −0.44, *r* = −0.19, and *r* = −0.31, respectively, all *p* < 0.01); job satisfaction (*r* = −0.48, *p* < 0.01); and, physical and mental health (*r* = −0.17 and *r* = −0.39, respectively, *p* < 0.01). Moreover, job insecurity (measured at T1) was significantly and positively related to turnover intentions at Time 2 (*r* = 0.42, *p* < 0.01). Finally, identifying with a greater number of marginalized identities was generally associated with more negative job- and health-related outcomes, including safety compliance (*r* = −0.11, *p* < 0.05), affective and normative commitment (*r* = −0.15 and *r* = −0.13, respectively, *p* < 0.01), job satisfaction (*r* = −0.16, *p* < 0.01), and physical and mental health (*r* = −0.13 and *r* = −0.21, respectively, *p* < 0.01).

### 3.2. Hypothesis Tests

In order to test our direct and indirect effects hypotheses, we used the SPSS PROCESS (IBM Corp., Armonk, NY, USA) macro (Model 4) created by Hayes [74] with intersectionality modeled as our independent variable, job insecurity as our single mediator variable, and the various outcomes as our dependent variables. In addition to providing regression coefficients for the specified paths, it also utilized N = 5000 samples to obtain bias corrected bootstrap confidence intervals of the effects.

Table 2 presents the results of these model tests examining the mediating role of job insecurity in the relationships between intersectionality and our outcomes of interest. As can be seen, in support of Hypothesis 1, intersectionality was significantly and positively associated with higher perceived job insecurity, B = 0.11, *t* (447) = 2.89, *p* < 0.01. Moreover, for Time 1, job insecurity significantly predicted Time 2 levels of safety motivation (B = −0.08, *p* < 0.01), safety compliance (B = −0.08, *p* < 0.01), affective commitment (B = −0.80, *p* < 0.01), continuance commitment (B = −0.24, *p* < 0.01), normative commitment (B = −0.41, *p* < 0.01), turnover intentions (B = 0.45, *p* < 0.01), job satisfaction (B = −0.81, *p* < 0.01), physical health (B = −0.11, *p* < 0.01), and mental health (B = −0.42, *p* < 0.01), providing support for Hypothesis 2.

Additionally, across all relationships examined, the 95% bootstrapped confidence intervals of the indirect effect estimates did not contain zero (see Table 2). Thus, job insecurity was a consistently significant explanatory mechanism accounting for the relationship between intersectionality and the outcomes of interest. Specifically, estimates of the indirect effect ranged in magnitude from −0.01 (for safety motivation and compliance) to −0.09 (for job satisfaction), thus providing support for Hypothesis 3.

Finally, even after accounting for the effects of job insecurity, intersectionality remained a significant predictor of: safety compliance, affective commitment, normative commitment, job satisfaction, and physical and mental health. This suggested that while job insecurity is a significant explanatory mechanism accounting for the effects of intersectionality on our outcomes of interest, intersectionality appeared to have direct effects on these variables as well. Alternatively, there may be other unexamined mediating mechanisms at play.

## 4. Discussion

Despite the ever-diversifying workforce of modern America, research has only begun to explore the unique impact of intersectionality on work-related outcomes. In particular, little work has inclusively incorporated the breadth of potential stigmatized identities in a comprehensive model, which may lead to under- or over-estimation of the impact of various identities intersecting. The current study aimed to explore whether intersectionality prompted the perception of job insecurity, a known economic stressor, among employees. Furthermore, this study assessed whether intersectionality indirectly impacted several of the known attitudinal, health, and safety-related negative outcomes of heightened job insecurity.

Together, our results showed that employees who identified with increasing numbers of stigmatized identities were also more likely to perceive and worry about their job insecurity. While much of the job insecurity literature has focused on delineating its outcomes (e.g., safety motivation, safety compliance, turnover intentions, job satisfaction, physical health, mental health, affective-, continuance-, and normative-commitment [4,6,7]), relatively little is known about what prompts the perception and development of job insecurity. While Keim and colleagues [9] provided an important meta-analytic review of individual and organizational antecedents, this work did not and could not look at the additive effects of holding multiple minority demographic characteristics. Therefore, we are one of the first to assess, and find support for, the relationship between identifying with greater stigmatized identities and increased perceived job insecurity. Furthermore, intersectionality was directly associated with many of the outcomes of interest, showing that intersectionality directly affected many other negative workplace outcomes, namely, safety compliance, affective commitment, normative commitment, job satisfaction, physical and mental health. Finally, the results indicated that the relationship between intersectionality and these harmful workplace outcomes were significantly explained by job insecurity perceptions as the intervening mechanism.

### 4.1. Theoretical Implications

In addition to enlarging our understanding of how demographic and other identity characteristics of employees may collectively impact their perceptions of job insecurity, our study also provides support for minority stress theory [46] by integrating arguments pertaining to intersectionality. We demonstrated that individuals with greater stigmatized identities experienced greater perceptions of a salient form of stress at work and job insecurity. Just as research has used minority stress theory to explain the workplace experiences of a double jeopardy or an intersection of two identities specifically [51,52], our study provides support that this carries on beyond two identities and pertains to intersectionality and the instances of more than just two minority identities. Indeed, as intersectionality increased, minority employees reported increasing experiences of stress, in particular, the economic stressor of job insecurity. In summary, the results of our study contribute to a growing body of work highlighting the unique workplace experiences of intersectional individuals and identify a meaningful antecedent to the well-established economic stressor of job insecurity, elucidating one element of stress contributing to the psychosocial stress that marginalized individuals inherently experience due to their minority status as the minority stress theory posits [46,47]. Furthermore, these findings are just one novel way that workforce diversity can influence occupational health. There are plenty of other opportunities for future research to explore the ways in which intersectionality may impact other aspects of occupational health, such as safety, financial strain, underemployment, and work-life conflict.

Second, our study combines two areas of research, which have largely run independently from each other, workplace diversity and occupational health. By understanding how intersecting identities may impact an employee’s health, safety, and well-being, we attempt to illuminate unique individual experiences, which impacts each worker as well as the organization which employs them. Future work could consider other ways in which the demographic makeup of the employee or potential mistreatment they may incur might impact areas of occupational health or workplace safety.

### 4.2. Practical Implications

Practically, this study utilized a novel approach to measuring intersectionality. This additive measure of intersectionality is a useful first approach or first step in exploring the potential impact of this unique variable. Once the impact of intersectionality is assessed, one could take their empirical exploration a step further by parsing out the specific intersections of interest, to determine what combinations of identities are having unique or differing experiences compared to other interacting identities. The research could potentially determine purposeful suggestions for policy and developing an inclusive organizational culture. Indeed, policy that speaks to just one dimension of one’s identity (i.e., policy focused on protecting just women or racial minorities) may not be enough to successfully attenuate workplace mistreatment—highlighting the need to consider intersectionality in organizational policy in accordance with past work [75].

The diversity of the workforce is increasing as time passes [10], and more intersectional employees are making up the working population. Stemming from this information, the results of this study and future work along this vein, human resource departments within organizations may want to strive for more inclusive policies (e.g., directly state more identity statuses in company policy statements). For example, Ragins and Cornwell [60] found that an organizational policy was one of the most important antecedents in preventing sexual orientation discrimination. This makes organizational policy changes an impactful way to prevent discrimination from occurring. The most influential ways to prevent discrimination because of one’s sexual orientation were the following: (1) including sexual orientation in the organization’s definition of diversity, (2) stating that the discrimination of individuals based on sexual orientation was not allowed or tolerated, (3) providing benefits for same-sex partners and (4) the inclusiveness of same-sex partners in organizational functions by inviting partners to social events [60]. While this pertains to sexual orientation specifically, we contend that this information is helpful for organizations that care to promote diversity and inclusion, and will be helpful for employees. While this example was specifically about sexual orientation discrimination, it highlights the potential that organizational policy can have in reducing discrimination and increasing inclusion within the workplace. Furthermore, organizations may want to prioritize their organizational culture and rectify any issues they already see to make it as inclusive towards a variety of identities. As seen in the headlines and popular press articles, some large organizations such as Starbucks have already made strides in retraining employees [76] after major discrimination occurrences. As minority stress theory states [46], minority individuals experience stress specifically stemming from their minority identity. Furthermore, as research on double jeopardy [51,52] as well as the current study showed, increased identities fall in line with this theory and result in additional psychosocial stress. Thus, it would behoove a company to take a preventative approach to reduce the impact of this psychosocial stress by focusing on the inclusion of various identities, so diverse workers feel genuinely welcome and aim to avoid adding any additional stress for these workers.

Lastly, as previous meta-analyses have indicated [4,6,7], job insecurity is a potent economic stressor, which leads to a host of negative outcomes pertaining to health, safety, turnover, and job satisfaction. Many instances of job insecurity are unavoidable, as organizations will go through mergers, restructuring and ultimately, layoffs. Thus, prevention is not the plausible option for workers in these cases. However, knowledge of which employees may be most at risk of experiencing job insecurity, particularly in these instances (e.g., layoffs) could allow for better targeting of organizational resources, such as resume workshops or how to emotionally cope with this economic stressor. Considering the results of the current study, organizations undergoing mergers or layoffs should consider the heightened job insecurity experienced by intersectional individuals and provide resources aimed at combating the known negative impacts of job insecurity.

### 4.3. Limitations and Future Directions

While this study presented intriguing findings that were novel, there is further potential impact on occupational health yet to be explored. The results of our study need to be interpreted while considering several limitations. Specifically, the study utilized an additive measure of intersectionality; exploring potential combinations of intersecting identities (i.e., a multiplicative approach) might yield further insights into the relationship between intersectional identities and perceptions of job insecurity. Nevertheless, the current approach allowed for an initial exploration of this relationship. Furthermore, this study is correlational; however, this study is a novel and important first step in understanding the unique relationships between intersectionality and economic stressors.

Now that intersectionality has been shown to be predictive of job insecurity and several of its known individual and organizational outcomes, one could take the next step in exploring the interactive impact of differing sets of identities (e.g., race and gender, sexual orientation and religion). For example, it could be that a few particular identities carry heavier weight in the relationship between intersectionality and job insecurity perceptions. Additionally, an important distinction comes from the visibility of different identity statuses in shaping the experiences of intersectional employees. More visible identities such as race or gender may intertwine in different ways as compared to more invisible identities such as sexual orientation or a veteran status. As such, future scholars should be mindful of identity variations such as visible, invisible, and visibly dynamic (e.g., pregnant workers, transgender workers), that may shape the ways in which employees experience work.

Another potential shortcoming may be our model specification, as we looked solely at one potential antecedent of job insecurity perceptions. While the purpose of this study was to test whether numerous different stigmatized/minority identities led to the perception of employee job insecurity, there were numerous other variables that may also have affected these perceptions (e.g., current organizational changes, prior employment/unemployment experiences, etc.) but were not measured in this study. Thus, there is the potential to mis-specify the relative importance of intersectionality in predicting job insecurity.

Nevertheless, while we restricted our focus to intersectionality, we used a methodologically rigorous approach to evaluate its direct and indirect effects on our outcomes of interest. For example, our use of longitudinal data considerably diminished the potential for multi-method bias which could ensue from cross-sectional survey studies. Additionally, longitudinal data improves causal inferences from a mediation analysis. Thus, while our study took a narrow focus on intersectionality as an antecedent of job insecurity, the results were strengthened by the research design.

## 5. Conclusions

In 2018, the workforce in the United States is arguably at its most diverse. Considering this, scholars and practitioners should move beyond examining identity in a singular manner; as employees are likely to be intersectional and identify with more than one stigmatized identity. Our study provides preliminary evidence that intersectionality (i.e., multiple intersecting stigmatized identities) is a risk factor for experiencing greater job insecurity and its sequelae, clearly playing an impactful role while navigating the modern workplace. Knowledge of which employees may be most at risk of job insecurity (particularly during times of restructuring or layoffs) could allow for a better targeting of organizational resources to combat its negative impacts. Future research might focus on whether certain minority identities or combinations thereof might be particularly predictive of job insecurity and other economic stressors.

## Figures and Tables

**Table 1:** Descriptive statistics and correlations.

Variable	M	SD	1	2	3	4	5	6	7	8	9	10	11	12
1. Intersectionality (T1)	2.46	1.19	n/a											
2. Job Insecurity (T1)	0.74	0.93	0.13 **	0.95										
3. Job Insecurity (T2)	0.70	0.88	0.12 *	0.84 **	0.95									
4. Safety Motivation (T2)	4.41	0.60	−0.05	−0.13 **	−0.08	0.90								
5. Safety Compliance (T2)	4.42	0.58	−0.11 *	−0.14 **	−0.15 **	0.71 **	0.91							
6. Affective Commitment (T2)	4.37	1.53	−0.15 **	−0.44 **	−0.46 **	0.18 **	0.19 **	0.92						
7. Continuance Commitment (T2)	3.30	1.20	−0.06	−0.19 **	−0.18 **	−0.08	−0.07	0.08	0.83					
8. Normative Commitment (T2)	3.70	1.30	−0.13 **	−0.31 **	−0.34 **	0.12 *	0.13 **	0.60 **	0.02	0.89				
9. Turnover Intentions (T2)	2.00	1.01	0.08	0.42 **	0.47 **	−0.16 **	−0.18 **	−0.70 **	−0.02	−0.42 **	0.88			
10. Job Satisfaction (T2)	5.26	1.60	−0.16 **	−0.48 **	−0.53 **	0.19 **	0.21 **	0.79 **	0.14 **	0.49 **	−0.78 **	0.94		
11. Physical Health (T2)	3.36	0.61	−0.13 **	−0.17 **	−0.22 **	0.00	0.01	0.19 **	0.26 **	0.06	−0.23 **	0.18 **	n/a	
12. Mental Health (T2)	4.45	1.06	−0.21 **	−0.39 **	−0.40 **	0.17 **	0.19 **	0.46 **	0.28 **	0.27 **	−0.43 **	0.50 **	0.48 **	0.95

Note: Listwise N = 444; * *p* < 0.05; ** *p* < 0.01. Cronbach’s alpha reliability coefficients are on the diagonal; variables indicated with n/a are formative (rather than reflective) constructs.

**Table 2:** The direct and indirect effects of intersectionality on outcomes.

			**Bootstrapped CI [95%]**	
	**Coeff**	**SE**	**LL**	**UL**
Intersectionality → Job Insecurity	0.11 **	0.04	0.03	0.18
	**Safety Motivation**			
			**Bootstrapped CI [95%]**	
	**Coeff**	**SE**	**LL**	**UL**
Job Insecurity → Safety Motivation	−0.08 **	0.03	−0.14	−0.02
Intersectionality → Safety Motivation	−0.02	0.02	−0.05	0.11
Indirect effect	−0.01 *	0.01	−0.02	−0.00
	**Safety Compliance**			
			**Bootstrapped CI [95%]**	
	**Coeff**	**SE**	**LL**	**UL**
Job Insecurity → Safety Compliance	−0.08 **	0.03	−0.14	−0.02
Intersectionality → Safety Compliance	−0.05 *	0.02	−0.09	−0.00
Indirect effect	−0.01 *	0.01	−0.02	−0.00
	**Affective Commitment**			
			**Bootstrapped CI [95%]**	
	**Coeff**	**SE**	**LL**	**UL**
Job Insecurity → Affective Commitment	−0.70 **	0.07	−0.84	−0.56
Intersectionality → Affective Commitment	−0.12 *	0.06	−0.23	−0.01
Indirect effect	−0.07 *	0.03	−0.13	−0.02
	**Continuance Commitment**			
			**Bootstrapped CI [95%]**	
	**Coeff**	**SE**	**LL**	**UL**
Job Insecurity → Continuance Commitment	−0.24 **	0.06	−0.36	−0.12
Intersectionality → Continuance Commitment	−0.03	0.05	−0.12	0.07
Indirect effect	−0.03 *	0.01	−0.05	−0.01
	**Normative Commitment**			
			**Bootstrapped CI [95%]**	
	**Coeff**	**SE**	**LL**	**UL**
Job Insecurity → Normative Commitment	−0.41 **	0.06	−0.53	−0.28
Intersectionality → Normative Commitment	−0.10 *	0.05	−0.20	−0.01
Indirect effect	−0.04 *	0.02	−0.08	−0.01
	**Turnover Intentions**			
			**Bootstrapped CI [95%]**	
	**Coeff**	**SE**	**LL**	**UL**
Job Insecurity → Turnover Intentions	0.45 **	0.05	0.35	0.54
Intersectionality → Turnover Intentions	0.03	0.04	−0.05	0.10
Indirect effect	0.05 *	0.02	0.02	0.09
	**Job Satisfaction**			
			**Bootstrapped CI [95%]**	
	**Coeff**	**SE**	**LL**	**UL**
Job Insecurity → Job Satisfaction	−0.81 **	0.07	−0.95	−0.66
Intersectionality → Job Satisfaction	−0.13 *	0.06	−0.24	−0.02
Indirect effect	−0.09 *	0.03	−0.15	−0.03
	**Physical Health**			
			**Bootstrapped CI [95%]**	
	**Coeff**	**SE**	**LL**	**UL**
Job Insecurity → Physical Health	−0.11 **	0.03	−0.14	−0.05
Intersectionality → Physical Health	−0.05 *	0.03	−0.05	−0.01
Indirect effect	−0.01 *	0.01	−0.02	−0.00
	**Mental Health**			
			**Bootstrapped CI [95%]**	
	**Coeff**	**SE**	**LL**	**UL**
Job Insecurity → Mental Health	−0.42 **	0.05	−0.51	−0.32
Intersectionality → Mental Health	−0.14 **	0.04	−0.22	−0.07
Indirect effect	−0.04 *	0.02	−0.08	−0.01

Note: * *p* < 0.05; ** *p* < 0.01; Coeff = coefficient; SE = standard error; LL = lower limit; UL = upper limit; intersectionality and job insecurity from Time 1; outcome variables from Time 2.
